# Human Liver Stem Cells: A Liver-Derived Mesenchymal Stromal Cell-Like Population With Pro-regenerative Properties

**DOI:** 10.3389/fcell.2021.644088

**Published:** 2021-04-26

**Authors:** Stefania Bruno, Maria Beatriz Herrera Sanchez, Giulia Chiabotto, Valentina Fonsato, Victor Navarro-Tableros, Chiara Pasquino, Marta Tapparo, Giovanni Camussi

**Affiliations:** ^1^Department of Medical Sciences, University of Torino, Turin, Italy; ^2^Molecular Biotechnology Center, University of Torino, Turin, Italy; ^3^2i3T, Società per la Gestione dell’incubatore di Imprese e per il Trasferimento Tecnologico, University of Torino, Turin, Italy

**Keywords:** hepatic stem cells, extracellular vesicles, acute liver injury, chronic liver disease, liver regeneration, renal regeneration, acute kidney injury, chronic kidney disease

## Abstract

Human liver stem cells (HLSCs) were described for the first time in 2006 as a new stem cell population derived from healthy human livers. Like mesenchymal stromal cells, HLSCs exhibit multipotent and immunomodulatory properties. HLSCs can differentiate into several lineages under defined *in vitro* conditions, such as mature hepatocytes, osteocytes, endothelial cells, and islet-like cell organoids. Over the years, HLSCs have been shown to contribute to tissue repair and regeneration in different *in vivo* models, leading to more than five granted patents and over 15 peer reviewed scientific articles elucidating their potential therapeutic role in various experimental pathologies. In addition, HLSCs have recently completed a Phase 1 study evaluating their safety post intrahepatic injection in infants with inherited neonatal onset hyperammonemia. Even though a lot of progress has been made in understanding HLSCs over the past years, some important questions regarding the mechanisms of action remain to be elucidated. Among the mechanisms of interaction of HLSCs with their environment, a paracrine interface has emerged involving extracellular vesicles (EVs) as vehicles for transferring active biological materials. In our group, the EVs derived from HLSCs have been studied *in vitro* as well as *in vivo*. Our attention has mainly been focused on understanding the *in vivo* ability of HLSC–derived EVs as modulators of tissue regeneration, inflammation, fibrosis, and tumor growth. This review article aims to discuss in detail the role of HLSCs and HLSC-EVs in these processes and their possible future therapeutic applications.

## Introduction

At present, it is estimated that 1.5 billion people are affected by chronic liver diseases that eventually progresses into fibrosis and cirrhosis (Moon et al., 2020). Liver transplantation currently represents the only efficient treatment that radically improves the outcome of liver failure. Despite efforts to expand the organ donor pool by extending the selection criteria and transplanting organs from related living donors, a lack of sufficient donors still remains the main issue to be solved (Adam et al., 2018). Although, transplanting of hepatocytes, obtained from adult or neonatal livers, is considered to be an alternative therapy to organ transplantation, and represents a potential treatment option also in patients with acute liver failure (Dhawan et al., 2010), a major limitation is the availability of organs for hepatocytes isolation as well as the difficulty to expand them *in vitro* (Squires et al., 2017). In this contest, the use of stem cells as a possible source of cells for hepatic reconstitution has emerged as a new potential therapeutic approach.

Human liver stem cells (HLSCs) were isolated for the first time in 2006, using a unique method based on stringent conditions of culture, whereby mature hepatocytes undergo cell death leaving clones of HLSCs that are easily expandable and exhibit multiple differentiating capabilities (Herrera et al., 2006). This discovery was considered to be innovative, leading to a patent approval in 2006 as a novel source of hepatic stem cells (WO2006126219A1, Liver progenitor cells).

The aim of this review is to describe HLSCs and their bio-products, as well as their potential effects in different therapeutic fields, such as regenerative medicine, oncology, and liver genetic diseases.

## HLSC Isolation and Characterization

HLSCs were isolated from both primary cultures and cryopreserved human hepatocytes cultured in stringent conditions (Herrera et al., 2006). They reached confluence within 3 weeks of culture and could be subcultured and expanded to large quantities for about 6 months without undergoing senescence (Herrera et al., 2006). For instance, HLSCs maintained in undifferentiating culture conditions for approximately 2–3 months with a lifespan of 200–250 doublings, retained remarkable stability demonstrated by the preserved telomere length during expansion (Bruno et al., 2019b).

**FIGURE 1:**
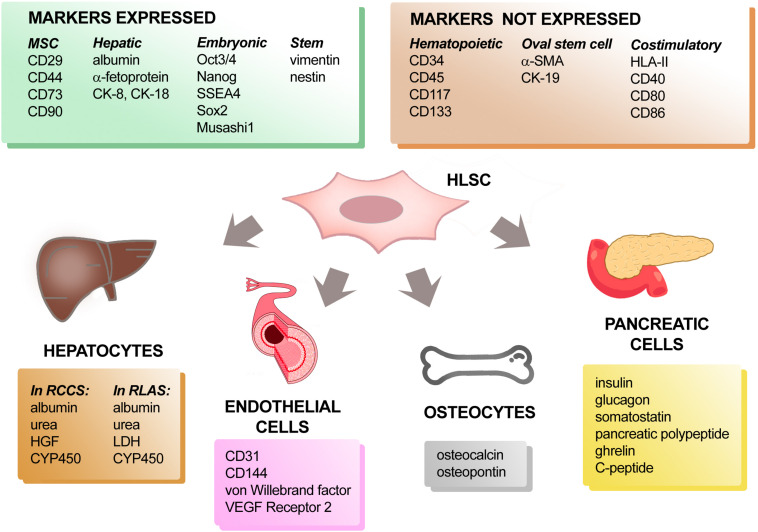
Phenotypic characterization and differentiation capacity of HLSCs. Protein markers expressed and not expressed by HLSCs are listed in the upper two panels. When cultured under appropriate conditions, HLSCs can differentiate into hepatocyte-like cells, endothelial cells, osteocytes, and pancreatic cells. The expression of tissue-specific markers acquired by differentiated-HLSCs is reported in the lower four panels.

HLSCs are negative for hematopoietic markers (CD34, CD45, CD117, and CD133), as well as human leukocyte antigen (HLA) class II and the costimulatory molecules such as CD40, CD80, and CD86 (Bruno et al., 2016). In addition, they express some mesenchymal stromal cell (MSC) markers (CD29, CD73, CD44, and CD90), together with, albumin, α-fetoprotein, cytokeratin (CK)-8 and CK-18 indicating a mesenchymal origin with partial commitment toward a hepatic lineage. They also express conventional stem cell markers such as vimentin and nestin and embryonic stem cell markers such as Oct3/4, Nanog, SSEA4, Sox2, and Musashi1 (Figure 1) which has been associated to self-renewal capacity and multipotency. However, unlike a well-known hepatic progenitor cell population, known as oval cells in rodents, HLSCs do not express CK-7, CK-19, c-kit, CD133 and alpha-smooth muscle actin (α-SMA) (Gaudio et al., 2009). The oval cells were described for the first time in the 1950s by Farber and colleagues and have oval-shaped nucleus and scant cytoplasm. In their experiments, Faber and colleagues observed mitosis in the small bile ducts and hyperplasia of the oval cells after ethionine diet (Farber, 1956). Subsequent studies suggested that the activity of these progenitor cells were mainly present at the canal of Hering and were involved in bi-potent activity, by replacing both damage hepatocytes and cholangiocytes (Wei et al., 2020). Although there are differences in the phenotypic markers between the HLSCs and oval cells, we can only speculate that HLSCs are a separate niche of stem cells in the liver, since it has not been confirmed *in vivo*.

The adult and fetal liver has been shown to harbor other stem cell populations that express some markers similar to HLSCs (Table 1). For instance, Najimi et al., described in 2007 the adult-derived human liver stem-like cells (ADHLSCs) which expressed both hepatic and mesenchymal markers, such as albumin and vimentin. However, their positivity for α-SMA and CD13 and not for CD105 (Najimi et al., 2007), suggested that ADHLSCs were different from HLSCs. An alternative approach reported by Lee et al., which involved the culturing of the non-parenchymal fraction of normal human adult liver (Lee et al., 2012), led to the isolation of HLSCs that expressed MSC-markers (CD29, CD73, CD44, CD90, CD105, and CD166, vimentin), nestin and hepatic markers (CD26, CK-8, and CK-18) (Lee et al., 2012). Moreover, Pan and colleagues described the presence of liver MSC-like population (L-MSCs) positive for CD90, CD105, and CD166 (Pan et al., 2011) in liver graft preservation fluid. The liver stromal cells (LSCs) described by Kellner et al. was another population of cells isolated and expanded from liver human biopsy, that expressed CD105, CD90, and CD73 (Kellner et al., 2015). Apart from adult human liver tissue, stem cells have also been isolated from fetal liver tissue. For instance, liver stem cells with MSC-characteristics and markers (CD29, CD73, CD44, CD90, CD105, CD166, and vimentin) were obtained from first- and second-trimester fetal livers (LHMSCs). These cells were positive for CD26, albumin, CK-8 and CK-18, therefore indicating a partial commitment toward hepatic differentiation (Campagnoli et al., 2001; In‘t Anker et al., 2003). In addition, Dan et al. identified another cell population from the fetal liver, known as human fetal liver multipotent progenitor cells (hFLMPCs). These cells co-expressed MSC (CD44, CD90, and vimentin), hematopoietic (CD34, c-kit), and cholangiocyte (CK-19) markers, as wells as EPCAM, c-met, SSEA4, and hepatocyte (CK-18) (Dan et al., 2006). These studies therefore indicate several progenitor/stem-cell like populations derived from human liver that share similarities and differences and exhibit regenerative properties, nonetheless whether these cells are localized in the liver or develop during *ex vivo* culture conditions remains to be elucidated (Table 1).

**TABLE 1:** Origin, markers and differentiation capabilities of liver MSC-like populations.

	**Origin**	**Expressed markers**	**Differentiation capabilities**	**References**
Human liver stem cells (HLSCs)	Frozen hepatocytes or derived from enzymatic digested liver biopsy from human adult donors	CD29, CD73, CD44, CD90, CD105, albumin, alfa-fetoproetein, CK-8 and 18, vimentin, nestin, Oct-3/4, nanog, SSEA-4, Sox-2, musashi	Osteocytes, endothelial cells, hepatocyte-like cells, islet-like structures	Herrera et al., 2006; Navarro-Tableros et al., 2019; Spada et al., 2020
Adult-derived human liver stem/progenitor cells (ADHLSCs)	Enzymatically digested adult human liver	CD29, CD73, CD44, CD90, CD13, HLA-class I, albumin, alfa-fetoprotein, CYP3A4, vimentin, α-smooth muscle actin	Hepatocyte-like cells	Najimi et al., 2007
Liver-derived mesenchymal stem cells (L-MSCs)	Graft preservation fluid collected from human liver graft	CD90, CD105, CD166,	Adipocytes and osteocytes	Pan et al., 2011
Human liver stem cells (HLSCs)	Non-parenchymal fraction of normal adult human liver	CD29, CD73, CD44, CD90, CD26, CD105, CD166, HLA-class I, vimentin, nestin, CK8, and 18	–	Lee et al., 2012
Liver stroma stem cells (LSCs)	Human liver biopsy	CD73, CD90, CD105	Adipocytes and osteocytes	Kellner et al., 2015
Liver-derived human mesenchymal stem cells (LHMSCs)	First- and second-trimester fetal liver	CD29, CD73, CD44, CD90, CD105, CD166, CD26, albumin, CK-8 and 18, vimentin and nestin.	Adipocytes, osteocytes and chondrocytes, hepatocyte-like cells	Campagnoli et al., 2001; In‘t Anker et al., 2003
Human fetal liver multipotent progenitor cells (hFLMPCs)	First- and second-trimester fetal liver	CD34, CD90, c-kit, EPCAM, c-met, SSEA-4, CK-18, CK-19, CD44, vimentin	Adipocytes, osteocytes, chondrocytes, endothelial cells, hepatocyte and bile duct-like cells	Dan et al., 2006

Similar to MSCs, HLSCs display immunomodulatory properties by inhibiting the activation of immune cells. In particular, since HLSCs constitutively expressed cyclooxygenase 1 and indoleamine 2,3-dioxygenase, they were able to reduce the proliferation of mitogen-stimulated T-lymphocytes in a dose-dependent manner. HLSCs also prevented the degranulation capacity of natural killer cells (NKs) and experiments with blocking antibody against HLA-G indicated that the inhibitory effect of HLSCs on NK activity was mediated by the release of soluble HLA-G. Moreover, HLSCs suppressed dendritic cell (DC) differentiation and maturation, and the inhibition of prostaglandin E2 significantly reverted the negative effect of HLSCs on DC differentiation (Bruno et al., 2016). In addition, ADHLSs (Lombard et al., 2019), LSCs and LHMSCs also displayed immunomodulatory effects (In‘t Anker et al., 2003; Kellner et al., 2015).

Recent studies have reported that it is possible to obtain and expand HLSCs in Good Manufacturing Practice (GMP). A Master Cell Bank (MCB) of HLSCs was generated in 2011 by an approved GMP facility (Areta International, Gerenzano, Italy), starting from a 10 to 15 mm liver fragment of a donor, according to standard criteria set by the Centro Nazionale Trapianti and the requirements of the Directive 2001/20/EC (Bruno et al., 2019b; Spada et al., 2020). All reagents in the manufacturing process of MCB were suitable for clinical use, checked against specifications to ensure they come from a qualified supplier according to European and Italian GMP standards. The quality of the product was tested for safety and potency, according to validated methods (Spada et al., 2020). Also, HLSCs produced in these conditions expressed MSC-markers (CD29, CD73, CD90, etc.), hepatocyte precursor marker (α-fetoprotein), mature hepatocyte protein (albumin), stem cell markers (vimentin and nestin) and embryonic stem cell markers (Oct3/4, Nanog, SSEA4, Sox2, and Musashi1). In addition, they were negative for the hepatic oval cell marker CK-19 and α-SMA (a marker of activated stellate cell) (Bruno et al., 2019b; Spada et al., 2020). Moreover, gene profiling of HLSCs with bone marrow derived MSCs using a specific MSC PCR array revealed HLSCs to exhibit a similar gene expression profile to MSCs. Only 13 out of 84 genes were differentially expressed in HLSCs with respect to MSCs. The gene expression profile also confirmed the presence of specific hepatic markers, such as hepatocyte growth factor (HGF) and CK-18 in HLSCs (Bruno et al., 2019b).

## HLSC Differentiation Abilities *in vitro*

A success in generating “neo-livers” represents one of the most important experimental strategies that may solve the lack of donors for liver transplantation (Saito et al., 2020). The ability of HLSCs to differentiate into functional hepatocyte-like cells has been reported using different *in vitro* or *ex vivo* systems (Figure 1). To obtain differentiation into hepatocytes, HLSCs were cultured both under adhesion and microgravity conditions in media supplemented with growth factors. In particular, HLSCs maintained for 15 days in the presence of HGF and fibroblast growth factor (FGF) 4 changed their morphology from elongated to cuboid cells. Expression of AFP was reduced and CK-8 and CK-18 expression increased. When cultured in a Rotary Cell Culture System (RCCS) in the presence of HGF and FGF-4, HLSCs differentiated into functional hepatocyte-like cells that expressed cytochrome P450 (CYP450) and were able to produce urea (Herrera et al., 2006; Fonsato et al., 2010). At variance with this finding, albumin was synthesized and released by HLSCs cultured both under adhesion and microgravity conditions (Herrera et al., 2006). These data indicated that the rotary system favored HLSC maturation into functional hepatocyte-like cells, since the same combination of growth factors did not permit differentiation into functional hepatocytes of HLSCs in 2D cultures. In addition, when cultured in a bio-artificial liver consisting of a filter of packed flow hollow fibers connected to a rotary bioreactor perfusion system, HLSCs lost their stem cell markers and acquired several properties like mature hepatocytes, such as secretion of albumin, urea and HGF, and increased expression of CYP450 isoenzymes compared to HLSCs maintained in adhesion culture conditions. Furthermore, in the rotary system, HLSCs acquired specific functions of mature hepatocytes, such as glucose consumption, CYP450 enzymatic activity, glycogen synthesis and incorporation/release of indocyanine green (Fonsato et al., 2010). In contrast, ADHLCSs already exhibited CyP3A4 enzymatic activity at a basal condition (Najimi et al., 2007) and differentiated efficiently into metabolically active hepatocyte like-cells in collagen I coated adhesion condition *in vitro* after sequential incubation with specific growth factors and cytokines (Khuu et al., 2011).

To generate a functional liver organ, it is important to recreate the native cell components (hepatocytes, endothelial and epithelial cells), together with the specific hepatic microenvironment (extracellular matrix components), able to give structural and functional support to the new organ. One of the most innovative experimental approaches is represented by technologies for xenotransplantation and tissue engineering that attempt to decellularize and recellularize the residual scaffold with the recipient cells (Rossi et al., 2019). Using rat liver acellular scaffolds (RLAS) as a biological support, HLSCs were able to differentiate into hepatocyte-like cells (Navarro-Tableros et al., 2015; Figure 1). They acquired the expression of mature hepatic markers, such as lactate dehydrogenase (LDH) and three subtypes of CYP450 with a parallel increased expression of albumin. Furthermore, the hepatocyte-like cells generated using RLAS were found to be metabolically active, as demonstrated by the presence of urea nitrogen in the conditioned medium. Additionally, the HLSC-recellularized RLAS showed to contain subgroups of cells located in the proximity, or properly attached, to the tubular remnant matrix structures. These cells expressed the cholangiocyte marker CK-19, the endothelial marker CD31 and the mesenchymal marker vimentin. All these observations support the ability of HLSCs to not only differentiate into mature hepatocyte-like cells, but also into epithelial-like and endothelial-like cells when seeded into natural liver scaffolds (Navarro-Tableros et al., 2015).

Therefore, interaction with matrix differential spatial distribution may drive specific HLSC differentiations reproducing at least in part, the liver structure. The differentiation of HLSCs into endothelial cells has been also obtained by maintaining HLSCs in the presence of vascular endothelial growth factor (VEGF) for up to 2 weeks (Figure 1). In this condition, HLSCs no longer expressed stem cells markers and started to express specific endothelial markers, such as CD31, CD144, von Willebrand factor and VEGF receptor 2 (Herrera et al., 2006).

Since liver and pancreas share common embryonic origins (Jennings et al., 2015), we investigated whether HLSCs have the ability to differentiate into pancreatic cells (Figure 1). After long term culture in the presence of high glucose and nicotinamide, HLSCs changed their morphology and started to form small spheroid cell clusters on top of the confluent cell monolayer. These three-dimensional cell clusters stained positively for human insulin and the glucose transporter Glut-2, indicating that HLSCs have the capacity to differentiate into islet-like structures (HLSC-ILSs) (Herrera et al., 2006). More recently, it was demonstrated that HLSCs could rapidly differentiate into insulin-producing 3D spheroidal cell aggregates through a one-step protocol based on charge-dependent aggregation, induced by protamine. The resulting HLSC-ILSs were 3D spheroidal cell organoids consisting of different cell subpopulations expressing insulin, glucagon, somatostatin, pancreatic polypeptide and ghrelin, just like α-, β- δ-, and ε-cells. Under basal conditions, HLSC-ILSs synthesized and secreted basal levels of insulin and human C-peptide exhibiting an immature phenotype (Navarro-Tableros et al., 2019). However, in the presence of high glucose and potassium concentrations, HLSC-ILSs were able to increase the levels of secreted C-peptide (Navarro-Tableros et al., 2019; Gomez et al., 2020). In an *in vivo* setting, when implanted under the kidney capsule of streptozotocin-diabetic immunodeficient mice, HLSC-ILSs significantly reduced hyperglycemia. Remarkably, a further phenotypic maturation occurred when HLSC-ILSs were implanted in diabetic mice, demonstrated by the upregulation of specific transcripts of beta-cell differentiation, such as PDX1 and NGN3 (Navarro-Tableros et al., 2019).

Similar to MSCs, HLSCs can also differentiate toward an osteogenic lineage (Figure 1). After 3 weeks of culture in osteogenic differentiation medium, the formation of a mineralized culture with alizarin red-positive calcium deposits indicated an osteogenic differentiation of HLSCs. These osteogenic differentiated HLSCs lost the expression of albumin, alfa-fetoprotein and CK-18 and gained the expression of bone-specific proteins, like osteocalcin and osteopontin. Unlike MSCs, no adipogenic differentiation abilities have been observed in HLSCs (Herrera et al., 2006). Taken together, these results indicate the pluripotency of HLSCs.

## Regenerative Capabilities of HLSCs *in vivo*

The first evidence that HLSCs can contribute to liver regeneration was reported in murine models of acute liver injury (ALI) (Herrera et al., 2006, 2013). Human cells were detected in murine hepatic tissues 30 days after injection of 2 × 10^5^ in immunodeficient mice with N-acetyl-p-aminophen-induced injury, indicating that HLSCs can integrate in regenerating livers (Herrera et al., 2006). This was further confirmed in a murine model of fulminant liver failure (FLF) induced by treating mice with intraperitoneal injections of D-galactosamine and lipopolysaccharide. Inoculation of HLSCs (2 × 10^6^) significantly increased mice survival and enhanced liver regeneration, reducing apoptosis and increasing proliferation of hepatic cells that survived the injury. In FLF murine model, liver cell localization was evaluated by IVIS. After intravenous injection, HLSCs preferentially accumulated in livers of mice with FLF but not in livers of healthy mice. Moreover, human cells were detected in the liver parenchyma of immuno-deficient mice as early as 7 days after injection. In addition, these cells, co-expressed CK-8 and CK-18, indicating differentiation of HLSCs into hepatocytes. Furthermore, an undifferentiated population of HLSCs still persisted in the murine hepatic tissue 21 days post inoculation. The overall effect observed following HLSC-treatment included improvement of liver function, as evaluated by reduced alanine and aspartate aminotransferases (ALT and AST) and ammonium plasma levels. Interestingly, the injection HLSC conditioned medium (HLSC-CM) had the same beneficial effects similar to the cells, indicating that paracrine factors produced by HLSCs could play a role in the observed pro-regenerative effect in limiting acute injury. The HLSC-CM contained several growth factors that could potentially be involved in liver regeneration, such as Interleukin (IL) 8 and 6, VEGF and HGF. In particular, treatment of mice with neutralizing anti-human HGF antibody abrogated the protective effect of HLSC-CM, signifying not only an important role of HGF toward the hepatoprotective effects of HLSC-CM, but also a paracrine based mechanism of action of HLSCs (Herrera et al., 2013). Similar results were also reported by Khuu et al., whereby ADHLSCs were able to accelerate hepatic regeneration and engraft *in vivo* after ALI (Khuu et al., 2013; Herrero et al., 2017).

The therapeutic effect of HLSCs was also evaluated in chronic liver disease (CLD), such as in a murine model of non-alcoholic steatohepatitis (NASH), induced by a diet deprived of methionine and choline (Bruno et al., 2019b). The injection of 1.5 × 10^6^ HLSCs at different time points of CLD development, not only induced an improvement of liver function and morphology, but also reduced inflammation and fibrosis, demonstrated by decreased expression of specific transcripts, such as Collagen I, α-SMA, transforming growth factor (TGF)-beta and IL-beta-1. Similar to acute models of liver injury, we also observed the presence of human cells in NASH mice 21 days post HLSC-injection as confirmed by histological and molecular analyses. Interestingly, in the NASH model, most of the human cells detected in the liver parenchyma did not express specific markers of hepatic commitment, suggesting that the differentiation of HLSCs into mature hepatocytes was not necessary to reduce liver fibrosis and inflammation. In a different experimental model of chronic liver disease induced by carbon tetrachloride, ADHLSCs also exhibited anti-fibrotic activity (Najimi et al., 2017). Furthermore, this effect was confirmed *in vitro* on hepatic stellate cells, whereby ADHLSCs and their CM inhibited their activation (Najimi et al., 2017).

Apart from Liver injury, HLSCs are also able to prompt tissue regeneration in various models of acute kidney injury (AKI). For instance, in an AKI mice model induced by intramuscular injection of glycerol, HLSCs (3.5 × 10^5^) were able to induce functional and morphological recovery of renal damage. Functional parameters such as blood urea nitrogen (BUN) and creatinine plasma levels were restored to near normal levels and a reduction of renal tubular necrosis and an increase of tubular cell proliferation was observed following treatment with HLSCs. In addition, similar to the lethal model of hepatic failure, treatment with HLSC-CM was also able to induce renal protection and regeneration in AKI, therefore indicating that paracrine factors produced by HLSCs also exhibit pro-regenerative effects on renal tissue (Herrera Sanchez et al., 2014).

HLSC-derived islet like organoids when implanted under the renal capsule significantly reduced hyperglycemia to normo-glycemia and restored the diabetic profile in SCID mice with streptozotocin-induced diabetes (Navarro-Tableros et al., 2019). In addition, these mice had detectable levels of human C-peptide indicating that the diabetes reversal observed could be due to transplantation of the islet like organoids. Furthermore, on comparing the gene profile of the HLSC-derived organoids transplanted *in vivo* to *in vitro*, the latter showed an immature gene expression profile compared to the former which underwent further β-cell differentiation becoming capable to reverse hyperglycemia in diabetic SCID mice.

## EVs Derived From HLSCs: Characterization

Among the different paracrine factors released by stem cells, their membrane derived vesicles emerged as an important mediator of their biological activity (Ratajczak et al., 2006). The potential application of stem cell-derived extracellular vesicles (EVs) in regenerative medicine has been investigated in the last years as an innovative approach. EVs are described as a heterogeneous population of membrane delimited particles released by all types of cells and present in different biologic fluids (Bruno et al., 2019a). EVs have been shown to exert a cell-to-cell communication function that can modify the behavior of target cells. This effect is relevant in both physiological and pathological conditions and is mediated through different mechanisms, such as direct signaling through receptor interaction or by transfer of their cargo that includes: several RNA species (mRNAs, miRNAs, long-non-coding-RNAs, tRNAs, rRNAs, circular-RNAs and piRNAs), proteins and bioactive lipids (Derkus et al., 2017). Stem cell-derived EVs may also induce epigenetic changes in injured recipient cells with the activation of regenerative programs (Camussi et al., 2010).

During the last few years, a lot of effort has gone into characterizing the HLSC-EV content to elucidate the role of the different components responsible for the effects observed *in vitro* and *in vivo*. HLSC-EVs displayed the typical shape and size of EVs, and the expression of typical exosomal marker such as CD9, CD81, and CD63. Moreover, the same surface markers as of the cell of origin were also observed on HLSC-EVs. In particular, they expressed high levels of CD29, CD44, CD105, and CD49e, whereas, CD142, CD146, SSEA4, and MCPS were expressed at a medium/low level. Hematopoietic (CD3, CD4, CD8, CD19), endothelial and epithelial markers were not detected on HLSC-EVs (Bruno et al., 2020).

Omic analysis revealed that HLSC-EVs contain RNA binding proteins, such as AGO2 and Alix (responsible for miRNA delivery), Staufen1 and 2 (involved in the transport and stability of mRNA), T-cell intracellular antigen-1 (TIA), TIA-1-related (TIAR), AU-rich element binding protein, as well as multifunctional proteins expressed in nuclei and stress granules. However, unlike stress granules, EVs did not contain the human ribosomal protein S29 (Collino et al., 2010; Iavello et al., 2016). Proteins involved in the formation of the endosomal sorting complex required for transport (ESCRT) were also described in HLSC EVs (Iavello et al., 2016) particularly Tsg101, CHMP4a, CHMP4b, and CHMP4c which were detected by western blot analysis. Protein array screening demonstrated the presence of many cytokines and transcription factors involved in the regulation of inflammation, p53 and PI3K pathways sustaining the anti-inflammatory effect exerted *in vivo* (Bruno et al., 2020).

Apart from proteins, different RNA species can also be delivered by EVs to target cells (Villarroya-Beltri et al., 2014). Microarray analysis demonstrated that HLSC-EVs shuttled a specific subset of mRNA involved in the control of transcription and metabolism. In particular, some detected gene transcripts are involved in cell proliferation such as MATK, MRE11A, CHECK2, MYH11, VASP, and CDK2 suggesting that HLSC-EVs could exert their pro-regenerative effect through the transfer of these transcripts (Herrera et al., 2010). Among the RNA species enriched in EVs, miRNAs have attracted a lot of interest because of their role in the regulation of gene translation (Bartel, 2004; Quesenberry et al., 2015). HLSC-EVs, has been shown to be enriched with more than a hundred miRNAs shared with the cell of origin, that have been shown to potentially regulate cell cycle, proliferation- and cell death program-related processes. Among these miRNAs, some are specifically shuttled by EVs and their targets contribute to the regulation of transcription and biosynthetic functions, such as macromolecule biosynthesis (Collino et al., 2010). Furthermore, HLSC-EVs vehicle miRNAs that can epigenetically modify target cells by inducing a pro-regenerative and anti-fibrotic program (Kholia et al., 2018; Grange et al., 2019).

## HLSC-EVs: *in vivo* and *ex vivo* Pro-Regenerative Capabilities

**FIGURE 2:**
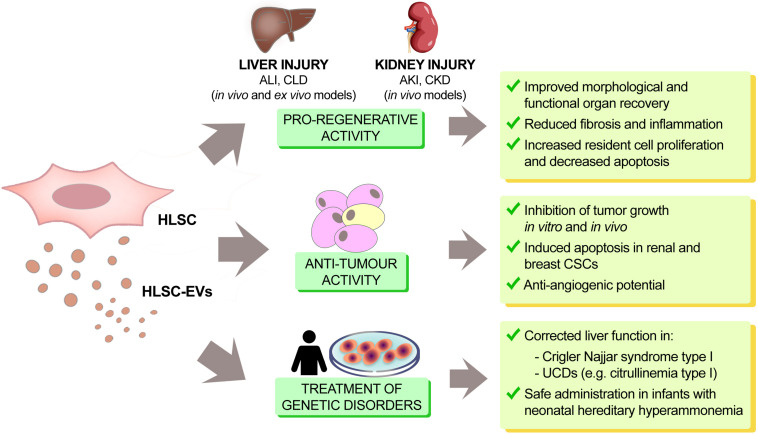
Therapeutic effects of HLSCs and HLSC-EVs. In renal and hepatic injury, HLSCs and HLSC-EVs act as modulators of tissue regeneration, inflammation and fibrosis, both *in vitro* and *in vivo*. They also display tumor suppressor potential in different types of cancers (hepatoma, lymphoblastoma, glioma, renal and breast carcinoma). HLSCs and their EVs can also restore proper enzymatic activity in liver genetic diseases (citrullinemia type I and Crigler Najjar syndrome type I). This fact, together with the demonstration that HLSC administration is safe in infants with hereditary hyperammonemia, paves the way for the possible use of HLSCs and HLSC-EVs in the treatment of genetic liver disease.

HLSC-derived EVs mimic the pro-regenerative effects of the cells of origin (Figure 2). For instance, HLSC-EVs were able to accelerate the morphological and functional recovery of the liver in an *in vivo* experimental model of ALI, consisting of partial a hepatectomy (70% hepatectomy in rats) (Herrera et al., 2010). This effect was associated with an increase in hepatocyte proliferation combined with a decrease in hepatocyte apoptosis, both of which were confirmed *in vitro.* Interestingly, this pro-regenerative effect was abrogated after pre-treatment of EVs with RNases, indicating an RNA-dependent effect through the horizontal transfer of RNA. These data therefore suggest that HLSC-EVs may activate a proliferative program in remnant hepatocytes post hepatectomy by a horizontal transfer of RNA. More recently, it has been reported that HLSC-EVs could contribute to improve hepatic function and morphology in NASH murine model of CLD. Administration of HLSC-EVs significantly ameliorated liver function as well as reduced fibrosis and inflammation at a histological level. In addition, the majority of fibrosis-associated genes up-regulated in NASH, were reverted following HLSC-EV treatment. Notably, bio-distribution experiments demonstrated that HLSC-EVs can preferentially accumulate in fibrotic liver (Bruno et al., 2020).

Similar to their cellular counterparts, HLSC-EVs as well ameliorated renal function and morphological damage in various murine models of both acute and chronic kidney injuries. For instance, HLSC-EVs were able to accumulate *in vivo* into damaged murine renal tubules and stimulate tubular cell proliferation, inducing an accelerated recovery of tubular damage. The pro-proliferative effects of HLSC-EVs has been confirmed also *in vitro*, on murine tubular epithelial cells (Herrera Sanchez et al., 2014). HLSC-EVs have been also tested in different murine models of chronic kidney disease (CKD). Non-immunocompetent mice treated with multiple injection of HLSC-EVs showed a significant reduction of histopathological signs of CKD and an amelioration of renal function. Progression of renal fibrosis that developed in diabetic nephropathy (DN) and aristolochic acid nephropathy (AAN) was significantly inhibited following HLSC-EV treatments. The expression levels of pro-fibrotic genes, such as Collagen I, α-SMA and TGF-beta, were significantly down-regulated in mice treated with HLSC-EVs (Kholia et al., 2018; Grange et al., 2019). Murine miRnome analysis of the kidneys of AAN mice treated with or without HLSC-EVs permitted the identification of miRNAs that were inversely correlated between the two treatment groups. Bio-informatic analyses identified more than 7,000 genes that were regulated by the miRNAs downregulated by HLSC-EVs, which were linked to about 140 pathways. Among them, the WNT signaling pathway and other inflammatory cytokines and chemokine pathways, such as TGF-beta, platelet derived growth factor (PDGF) and FGF pathways have been linked to the development of fibrosis (Kholia et al., 2018). In addition, HLSC-EVs shuttled a specific subset of miRNAs and bioinformatics analyses have indicated that they may act on pro-fibrotic pathways such as TGF-beta, insulin growth factor-1 (IGF-1), epidermal growth factor (EGF) receptor and PDGF receptor, consistent with their therapeutic effects on CKD (Grange et al., 2019). Some of the miRNAs enriched in HLSC-EVs include miRNA-29a, let-7 family, miRNA-30a, miRNA-24 and miRNA-21, which are known to directly target pro-fibrotic genes such as Collagen I (Schug et al., 2013; Balakrishnan et al., 2014), Snail57 and the FAS ligand (Sayed et al., 2010). Therefore, downregulation of pro-fibrotic genes, modulation of miRNAs that regulate the fibrotic pathways and shuttling of specific miRNA-subsets could be mechanisms through which HLSC-EVs exert their anti-fibrotic effects in CKD.

In order to elucidate the anti-fibrotic mechanism of HLSC-EVs, an *in vitro* model of CKD, that involved co-culturing aristolochic acid injured mTECs with mouse kidney cortical fibroblasts (mkCF), was adopted (Kholia et al., 2018). Treatment with HLSC-EVs inhibited upregulation of pro-fibrotic genes such as Collagen I, α-SMA and TGF-beta as was observed *in vivo*. Further analysis revealed HLSC-EV treatment downregulated five miRNAs in injured mkCF which have also been linked with CKD. In particular, miR-377-3p shown to be upregulated in diabetic nephropathy and exacerbates fibronectin production (Wang et al., 2008).

Preconditioning of the liver with HLSC-EVs may be an interesting option to limit tissue damage due to ischemia-reperfusion injury (IRI). In fact, the current method of organ preservation in static cold storage is unable to fully protect suboptimal livers from IRI. Normothermic machine perfusion (NMP) is emerging as a preservation technique potentially able to improve liver transplantation outcomes using extended criteria grafts (Butler et al., 2002; Imber et al., 2002; Brockmann et al., 2009). The physiological temperature together with the active hepatic metabolism in NMP permitted pharmacological intervention to ameliorate liver quality before transplantation (Goldaracena et al., 2017). The addition of HLSC-EVs during NMP contributed to the reduction of histological damage, apoptosis, and molecular over-expression of both hypoxia-inducible factor1-α and TGF-beta 1, indicating that HLSC-EV treatment during NMP reduced liver injury (Rigo et al., 2018). More recently, HLSC-EVs have been demonstrated to reduce liver damage in a murine model of IRI obtained by selective clamping of intrahepatic pedicles for 90 min followed by 6 h of reperfusion. The intravenous administration of HLSC-EVs attenuated hepatic IRI by significantly reducing necrosis, the release of transaminases and LDH, as well as the expression of pro-inflammatory cytokines TNF-α, chemokine (C-C motif) ligand 2 (CCL-2) and chemokine (C-X-C motif) ligand 10 (CXCL-10) (Calleri et al., 2020).

## HLSCs and HLSC-EVs: Anti-Tumor Activity

Paracrine factors released by embryonic stem cells (ESCs) can inhibit tumor growth (Postovit et al., 2008; Giuffrida et al., 2009). Similarly, bioproducts released by HLSCs have been described to be able to inhibit tumor growth *in vitro* and *in vivo* (Figure 2). In an *in vivo* setting, the intra-tumor administration of HLSC-CM inhibited the growth of HepG2 hepatoma cells implanted subcutaneously in SCID mice and in an *in vitro* setting, HLSC-CM not only inhibited the growth but also promoted apoptosis of HepG2 cells (Cavallari et al., 2013). HLSCs released Lefty A, an inhibitor of the Nodal signaling that is one of the mediators of anti-tumor effect exhibited by ESCs (Topczewska et al., 2006). HLSC-CM derived from Lefty A-silenced HLSCs was unable to inhibit tumor growth. This result indicated that Lefty A released by HLSCs may account, at least in part, for the tumor suppressive activity of HLSC-CM, as described for ESCs.

HLSC-EVs have also been reported to exert anti-tumor activities *in vitro* and *in vivo*. Intra-tumor injection of purified HLSC-EVs inhibited the growth of HepG2 cells *in vivo* and miRNAs shuttled by HLSC-EVs could be involved in their anti-tumor activity. Among them, miR451, miR223 and miR31 were shown to act as tumor suppressors, when transferred to HepG2 and primary hepatocarcinoma cells by HLSC-EVs (Fonsato et al., 2012). The involvement of these miRNAs in tumor progression is widely documented in different types of neoplastic pathologies (Nan et al., 2018; Bai and Wu, 2019; Dou et al., 2019; Jeffries et al., 2019; Mamoori et al., 2019).

The anti-tumor effect of HLSC-EVs is not specific for hepatoma cells, since this phenomenon has also been described on tumor cells derived from other cancers, such as lymphoblastoma and glioma (Fonsato et al., 2012). Interestingly, a tumor suppressor ability of HLSC-EVs has been demonstrated on Cancer Stem Cells (CSCs), known to sustain the initiation, maintenance, and recurrence of tumors. Furthermore, HLSC-EV treatment can induce apoptosis both in renal and breast CSCs (Fonsato et al., 2018). The mechanism underlying the anti-tumor activity exerted on CSCs by HLSC-EVs, alone or in combination with several Tyrosine Kinases Inhibitors (TKIs), may be ascribed to a modulation of PI3K and Erk pathways. The involvement of the PI3k/Akt/mTOR pathway in inhibition of cancer growth is well documented (Lv et al., 2018; Nan et al., 2018; Slattery et al., 2018). In particular, the inhibition of Akt and mTOR pathways and of PTEN with consequent inhibition of the tumor cell proliferation and induction of apoptosis was the result of the synergism of HLSC-EVs with TKIs. This synergism was evident using HLSC-EVs and low doses of TKIs and might increase the response to TKIs on CSCs, thus providing the proof of concept for a combined use of these products in the treatment of renal carcinoma.

The anti-tumor effect of HLSC-EVs has also been demonstrated *in vivo* in a model of renal carcinoma obtained by subcutaneous injection of renal CSCs. In this model, the intravenous administration of HLSC-EVs affected tumor development and growth and improved lung tumor-free survival (Brossa et al., 2020). The anti-tumor activity of HLSC-EVs was observed *in vitro* through the reduction of CSC invasion and organization into spheres, a stem-related characteristic, and also *in vivo* through the inhibition of tumor growth as well as tumor angiogenesis. The transfer of miR-145 and the induction of miR-200b and miR-200c transcription were shown to possess a pivotal role in impairing the functions of renal CSC (Brossa et al., 2020). HLSC-EVs also showed anti-angiogenic potential by impairing tumor endothelial cells to form capillary-like structures *in vitro* and tumor vessels *in vivo.* Furthermore, it was observed that the anti-angiogenic effect of HLSC-EVs was mediated by the transfer of specific anti-angiogenic miRNAs (Lopatina et al., 2019).

All these recent findings suggest a possible use of HLSC-EVs as a feasible alternative in cancer treatment to enhance efficacy of current therapies.

## HLSCs and HLSC-EVs: Tools to Treat Genetic Disorders

The administration of HLSCs could be a very promising approach to correct liver function in monogenic liver diseases (Figure 2). In fact, the efficacy of HLSCs was tested in a murine model of Crigler Najjar syndrome type I monogenic disease, caused by deficiency in uridine-diphosphate-glucuronosyltransferase (UGT1A1), the enzyme responsible for bilirubin conjugation in the liver. Following intra-parenchymal injection, HLSCs showed regional and heterogeneous engraftment in the injected lobe. Additionally, the expression of UGT1A1 in the liver of HLSC-injected mice increased, demonstrating that HLSCs were able to partially correct the deficiency of the activity of this enzyme (Famulari et al., 2020).

Liver transplantation has been used for the treatment of inborn errors of metabolism with a 95% success rate of children achieving long-term survival. However, liver transplantation cannot be routinely performed in newborns, especially for the high perioperative mortality at this age. Several studies indicated potential use of liver cell transplantation as a bridge therapy to correct the inherited enzyme deficiency with transient metabolic effectiveness (Lee et al., 2018). However, several technical limitations as well as availability of liver cells led to an increased interest in the potential use of stem cells with hepatic differentiation capability. The potential differentiation of HLSCs into mature hepatocytes candidate these cells for treatment of inherited enzyme deficiencies. In particular, the possibility of using HLSCs as an alternative cell-based therapy in genetic/metabolic diseases has been recently demonstrated by a clinical Phase I study in infants (Spada et al., 2020). HLSCs obtained by the European Medical Agency (EMA) in 2012 the designation of Orphan Drug for the treatment of rare diseases (EU/3/12/971 carbamoyl-phosphate synthase-1 deficiency; EU/3/11/904 ornithine transcarbamylase deficiency). The regulatory agency AIFA (Agenzia Italiana del Farmaco) authorized a first-in-man phase I clinical trial (HLSC 01–11, EudraCT-No. 2012–002120-33) as an open-label, prospective, uncontrolled, monocentric study, for the treatment of a group of rare genetic disorders in pediatric patients with neonatal hereditary hyperammonemia due to errors of metabolism, known as urea cycle disorders (UCDs). The study was conducted at the Liver Transplant Center of the University Hospital City of Health and Science (Azienda Ospedaliero-Universitaria Città della Salute e della Scienza di Torino) in Turin, Italy. This clinical trial started in January 2014 and ended in December 2016 and highlighted how the percutaneous intrahepatic administration of HLSCs was safe and well tolerated in neonates with hereditary hyperammonemia. The Phase 1 clinical study enrolled 3 neonates, without the administration of any immunosuppressive drug, to determine the safety of the cells *in vivo* and to evaluate hepatic and extra-hepatic complications after the liver intraparenchymal injection of HLSCs. Neonates received thawed HLSCs derived from frozen bags. Thawed cells were washed to reduce dimethyl sulfoxide concentration and doses were adjusted on the base of neonate body weight. During and after the administration of HLSCs, the patients were monitored continuously for temperature, heart and respiratory rates, blood pressure, and oxygen saturation, and the liver parenchyma was monitored by echography. At the end of the observation period, none of the patients showed sign of infections, hyperammonemia decompensation, intrahepatic or extrahepatic complications or other adverse events. Moreover, despite an increase (∼30%) in protein intake, all treated patients were metabolically stable. After explantation, the native livers of two patients that underwent liver transplantation (after 19 and 11 months, respectively) showed no histological alterations. These data confirmed the primary outcome of the study, which was the safety of HLSCs (Figure 2).

ADHLSCs were also approved for a Phase I/II clinical trial in pediatric liver-based metabolic disorders (F. Smets et al., 2019). The results of this study indicated the tolerability of the administration of stem cells via the portal vein. The adverse events observed in this study were in line with expectations for catheter placement, cell infusion, concomitant medications, age, and underlying diseases (J. Häberle et al., 2012). In Table 2 are listed the approved clinical trials of liver stem cells for liver disease.

**TABLE 2:** Summary of clinical trials of liver stem cells for liver disease.

**Title**	**Status**	**NCT number**	**Cell source**	**Desease**	**Link**
Safety study of HepaStem for the treatment of urea cycle disorders (UCD) and Crigler-najjar syndrome (CN)	Completed	NCT01765283	HHALPC, heterologous human adult liver derived progenitor cells	Urea cycle disorders Crigler najjar syndrome	https://ClinicalTrials.gov/show/NCT01765283
Human fetal liver cell transplantation in chronic liver failure	Completed	NCT01013194	Human fetal liver cell transplantation. Cell source: Non-purified and non-selected fetal liver cells from fetuses aborted between the 16 and 26th week of gestation.	Liver cirrhosis	https://ClinicalTrials.gov/show/NCT01013194
Improvement of liver function in liver cirrhosis Patients after autologous mesenchymal stem cell injection:a Phase I-II clinical trial	Completed	NCT00420134	Progenitor of hepatocyte drived from Mesenchymal stem cell	Liver failure cirrhosis	https://ClinicalTrials.gov/show/NCT00420134

HLSC-EVs were investigated as a possible therapeutic approach to correct genetic liver diseases (Figure 2). As the urea cycle takes place primarily in the liver, and HLSCs are of liver origin, the possible application of HLSC-EVs in the correction of UCDs was specifically evaluated in citrullinemia type I caused by a deficiency in arginosuccinate synthase-1 (ASS-1), one of the enzymes involved in the urea cycle. It has been demonstrated that HLSC-EVs carry and transfer the wild-type version of ASS-1 to ASS-1-mutated HLSCs *in vitro*, thereby restoring enzymatic activity and urea production in HLSC derived from a patient with the inherited deficiency in arginosuccinate synthase-1. ASS-1-mutated HLSCs were obtained from a small biopsy of the discarded liver from a transplanted patient with citrullinemia type I. The mutated HLSCs were characterized by cytofluorimetric analysis to confirm typical HLSC markers. SNaPshot sequencing of mutated HLSCs revealed that ASS-1 carried two codon mutations with the substitution of C and G bases with T and A (g.55277 C > T and g.59839 G > A), which affected the functional aspect of the enzyme. Furthermore, the exchange of the wild-type ASS-1 protein was able to restore the enzymatic activity of ASS-1 to basal levels in hepatocytes derived from differentiated ASS-1-mutated HLSCs treated with purified EVs from wild-type HLSCs (Herrera Sanchez et al., 2017).

To further study the contribution of HLSC-EV enriched with ASS-1 mRNA, HLSCs were transiently transfected with ASS-1-shRNA. EVs isolated and purified from the transfected cells were analyzed by an *in vitro* ASS-1 enzymatic assay to confirm the absence of ASS-1 protein inside the EVs. Unlike normal HLSC-EVs, the EVs from ASS-1-silenced HLSCs were unable to restore urea production in hepatocytes differentiated from mutated ASS-1-HLSCs, thus suggesting that the restoration mechanism may depend on the transfer not only of the protein but also of the intact functional ASS-1 mRNA (Herrera Sanchez et al., 2017).

The direct delivery of pure mRNA has also been shown to be effective for the treatment of urea cycle deficiencies. For instance, a group studying Ornithine Transcarbamylase (OTC) deficiency successfully normalized blood ammonia and improved the survival of OTC-deficient mice following direct administration of human OTC mRNA (Prieve et al., 2018). Although this direct delivery of mRNA has some advantages (Soria et al., 2019; Trepotec et al., 2019), one major limitation is the instability and relative short half-life of the mRNA *in vivo* (Soria et al., 2019; Trepotec et al., 2019). This limitation is not applicable to EVs, since they are membrane bound particles, and the cargo enriched within them, including mRNAs and proteins, is not only very well protected from degradation, but also display a prolonged biological activity *in vivo*, therefore making them suitable candidates for genetic disorders therapy (Bruno et al., 2019a; Meng et al., 2020).

## Conclusion

Cell therapy is an alternative form of treatment for liver disease that involves *in vitro* amplification followed by transplantation of healthy stem/progenitor cells into patients, ideally through a minimally invasive procedure (Wei et al., 2020). Currently, many clinical trials of stem cell-based therapies for liver diseases have been carried out or are ongoing, including MSCs, hematopoietic stem cells, and other bone-marrow derived cells (Wei et al., 2020). Nonetheless, several issues need to be taken into consideration in order to obtain this goal: firstly, it is essential that the cells remain genetically stable and non-tumorigenic as they proliferate; secondly, a sufficient number of healthy cells is required and thirdly, the cells need to maintain their properties under storage conditions.

Other sources of stem cells have also been identified with therapeutic properties, but also exhibited negative aspects that prevent their potential use in therapy. For instance, embryonic stem cells have the ability to proliferate robustly, as well as the capability to differentiate into functioning hepatic progenitor cells: however, ethical issues related to their source of origin together with their tumorigenicity remains a hindrance (Rao, 2007). Lately, the differentiation of human inducible pluripotent stem cells into hepatocyte-like cells have come to light, even though obtaining fully functional hepatocytes through this method has been proven difficult, as the whole process of differentiation contains several steps that influence the formation of hepatocytes in a negative way and requires further refinement (Cai et al., 2007).

The efficacy of MSCs in acute and chronic liver injury has been demonstrated in many animal experimental models. However, some potential risks and adverse effects in the application of MSCs in the treatment of liver diseases have been identified. For example, MSCs have the potential to differentiate into hepatic stellate cells and myofibroblasts, therefore promoting hepatic fibrosis (Yang et al., 2012). In addition, there is also the possibility of malignant transformation of MSCs following transplantation (Wu and Chen, 2006).

We have reviewed the properties of HLSCs based on our investigations developed in our laboratory for more than a decade. Nonetheless, further studies are still needed to understand their mechanisms of action. HLSCs have a versatile cell plasticity reflected by their multiple differentiation capabilities *in vitro*, therefore making them an eligible source for cell-based therapies. In addition, bio-distribution experiments in different *in vivo* models indicated the ability of HLSCs to reach the injured liver. Once in the target organ, HLSCs were able to engraft, as shown in acute and chronic liver injuries, whereby they were detected as cells of human origin 21 days after their injection (Herrera et al., 2006, 2013; Bruno et al., 2019b). Moreover, they were also found to be differentiated into hepatocytes expressing CK8 and CK18, even if an undifferentiated population of human cells persisted in a mouse model of acute liver injury (Herrera et al., 2013). Furthermore, in a NASH model of chronic liver injury, we demonstrated the presence of human cells that did not express markers of hepatocyte differentiation (Bruno et al., 2019b). These data indicated that the beneficial effects observed *in vivo* did not depend on the differentiation of HLSCs, suggesting that the bio-product of HLSCs could mediate the beneficial effects of the cells. Experiments where conditioned medium of HLSC was administered, confirmed that paracrine factors were involved in the pro-regenerative and anti-tumorigenic properties of the cells (Cavallari et al., 2013; Herrera et al., 2013). Among the paracrine effectors present in the conditioned medium, EVs were investigated thoroughly. We showed through multiple acute and chronic *in vivo* models of hepatic and renal injuries that HLSC-EVs exhibit pro-regenerative and tissue-protective effects (Herrera et al., 2010; Herrera Sanchez et al., 2014; Kholia et al., 2018; Grange et al., 2019; Bruno et al., 2020). These experiments support the idea that cell-free therapy could be an alternative approach for the treatment of different acute and chronic diseases. Compared with cell-based therapy, treatment with EVs has some advantages. EVs exhibit a superior efficacy profile as they pass biological barriers and act as carriers of different molecules (RNAs, proteins, and lipids). A significant benefit of EVs with respect to cell treatment is the chance to avoid potential tumorigenicity. *In vitro* and *in vivo* experiments indicated that HLSC-EVs could not only inhibit tumor growth but also increase the efficacy of anti-neoplastic drugs in a synergistic way (Fonsato et al., 2012, 2018; Brossa et al., 2020). Despite the effort to study EVs and their interactions with the microenvironment through several preclinical models, several aspects still have to be addressed before introducing them to a clinical setting. These include upscale production in a GMP format, characterization, pharmacokinetics, pharmacodynamics, toxicity and host immune reaction among others. Moreover, EVs from various stem cell sources (embryonic, adult, bone marrow, umbilical cord) have been tested in different *in vitro* and *in vivo* models of disease. A direct comparison of HLSC-EVs with EVs derived from other stem cells sources in liver disease models is still missing. Among the different sources, human umbilical cord-MSCs could be one of the best cellular sources, because accessible and not involving ethical objections (Ifrah et al., 2020).

Despite the multiple studies that have described the possible role of liver stem cells and EVs in liver regeneration, their contribution toward liver hemostasis is still under debate (Groeneveld et al., 2019; Michalopoulos and Bhushan, 2021). Recently, Groeneveld et al. identified a new mechanism whereby coagulation-dependent intrahepatic fibrin(ogen) deposition leads to the accumulation of platelets and therefore activates liver regeneration after partial hepatectomy. To our knowledge, the contribution of stem cells and their EVs toward this new mechanism has not been elucidated and therefore further studies are required.

In conclusion, HLSCs have shown to be a particular cellular entity that interacts with other cells locally and systemically to provide cell-based responses with several therapeutic properties. Much remains to be gained in terms of scientific knowledge and clinical benefit as the complex biology and therapeutic potential of HLSCs are continuously under development.

## Author Contributions

SB, MH, GCh, VF, VN-T, CP, and MT performed the research of the pertinent literature, designed and drafted the manuscript. GCa revised and edited the manuscript. All authors contributed to the article and approved the submitted version.

## Conflict of Interest

GCa was a component of the scientific advisory board of Unicyte AG. SB, MH, VF, VN-T, and GCa were named inventors in related patents. The remaining authors declare that the research was conducted in the absence of any commercial or financial relationships that could be construed as a potential conflict of interest.

## Abbreviations

AAN: aristolochic acid nephropathy
ADHLSCs: adult-derived human liver stem-like cells
AKI: acute kidney injury
ALI: acute liver injury
ALT: alanine aminotransferase
AST: aspartate aminotransferase
AIFA: Agenzia Italiana del Farmaco
ASS-1: argininosuccinate synthase-1
BUN: blood urea nitrogen
CYP450: cytochrome P450
CK: cytokeratin
CCL-2: chemokine (C-C motif) ligand 2
CXCL-10: chemokine (C-X-C motif) ligand 10
CSCs: cancer stem cells
CKD: chronic kidney disease
DN: diabetic nephropathy
CLD: chronic liver disease
DC: dendritic cells
ESCs: embryonic stem cells
EVs: extracellular vesicles
EMA: European Medical Agency
EGF: epidermal growth factor
ESCRT: endosomal sorting complex required for transport
FGF: fibroblast growth factor
FLF: fulminant liver failure
GMP: good manufacturing practice
HGF: hepatocyte growth factor
HLSCs: human liver stem cells
HLSC-ILSs: HLSC-islet like structures
hFLMPCs: human fetal liver multipotent progenitor cells
HLA: human leukocyte antigen
HLSC-CM: HLSC conditioned medium
IL: Interleukin
IGF-1: insulin growth factor-1
IRI: ischemia-reperfusion injury
L-MSC: liver MSC-like population
LHMSCs: liver human MSCs
LDH: lactate dehydrogenase
MCB: master cell bank
MSC: mesenchymal stromal cell
NKs: natural killer cells
NASH: non-alcoholic steatohepatitis
NMP: normothermic machine perfusion
OTC: ornithine transcarbamylase
PDGF: platelet derived growth factor
RCCS: rotary cell culture system
RLAS: rat liver acellular scaffolds
TGF: transforming growth factor
mkCF: mouse kidney cortical fibroblast
TIA-1: T cell internal antigen 1
TIAR: TIA-1 related
UGT1A1: uridine-diphosphate-glucuronosyltransferase
UCDs: urea cycle disorders
VEGF: vascular endothelial growth factor
α-SMA: alpha smooth muscle actin.
